# High Seroprevalence of HBV and HCV Infection in HIV-Infected Adults in Kigali, Rwanda

**DOI:** 10.1371/journal.pone.0063303

**Published:** 2013-05-22

**Authors:** John Rusine, Pascale Ondoa, Brenda Asiimwe-Kateera, Kimberly R. Boer, Jean Marie Uwimana, Odette Mukabayire, Hans Zaaijer, Julie Mugabekazi, Peter Reiss, Janneke H. van de Wijgert

**Affiliations:** 1 Department of Global Health, Academic Medical Center, and Amsterdam Institute of Global Health and Development (AIGHD), Amsterdam, The Netherlands; 2 INTERACT Program, Kigali, Rwanda; 3 National Reference Laboratory, Kigali, Rwanda; 4 Biomedical Research, Royal Tropical Institute, Amsterdam, The Netherlands; 5 Kigali University Teaching Hospital (KUTH), Kigali, Rwanda; 6 Department of Medical Microbiology, Academic Medical Center, Amsterdam, The Netherlands; 7 Institute of Infection and Global Health, University of Liverpool, Liverpool, United Kingdom; University of Cincinnati College of Medicine, United States of America

## Abstract

**Background:**

Data on prevalence and incidence of hepatitis B virus (HBV) and hepatitis C virus (HCV) infection in Rwanda are scarce.

**Methods:**

HBV status was assessed at baseline and Month 12, and anti-HCV antibodies at baseline, in a prospective cohort study of HIV-infected patients in Kigali, Rwanda: 104 men and 114 women initiating antiretroviral therapy (ART) at baseline, and 200 women not yet eligible for ART.

**Results:**

Baseline prevalence of active HBV infection (HBsAg positive), past or occult HBV infection (anti-HBc positive and HBsAg negative) and anti-HCV was 5.2%, 42.9%, and 5.7%, respectively. The active HBV incidence rate was 4.2/1,000 person years (PY). In a multivariable logistic regression model using baseline data, participants with WHO stage 3 or 4 HIV disease were 4.19 times (95% CI 1.21–14.47) more likely to have active HBV infection, and older patients were more likely to have evidence of past exposure to HBV (aRR 1.03 per year; 95%CI 1.01–1.06). Older age was also positively associated with having anti-HCV antibodies (aOR 1.09; 95%CI 1.04–1.14) while having a higher baseline HIV viral load was negatively associated with HCV (aOR 0.60; 95% CI 0.40–0.98). The median CD4 increase during the first 12 months of ART was lower for those with active HBV infection or anti-HCV at baseline. Almost all participants (88%) with active HBV infection who were on ART were receiving lamivudine monotherapy for HBV.

**Conclusion:**

HBV and HCV are common in HIV-infected patients in Rwanda. Regular HBsAg screening is needed to ensure that HIV-HBV co-infected patients receive an HBV-active ART regimen, and the prevalence of occult HBV infection should be determined. Improved access to HBV vaccination is recommended. Active HCV prevalence and incidence should be investigated further to determine whether HCV RNA PCR testing should be introduced in Rwanda.

## Introduction

In sub-Saharan Africa, 65–98% of the population will have lifetime exposure to hepatitis B virus (HBV) and 8–20% will become a chronic carrier [1]. The predominant modes of transmission are perinatal and horizontal (in early childhood), but transmission via unprotected sexual intercourse or intravenous drug use in adults also occurs [2], [3]. The proportion progressing from acute to chronic HBV is primarily determined by the age at infection: approximately 90 percent for perinatal infection, 20–50 percent for early childhood infection, and less than 5 percent for adult infection [4]. Hepatitis C virus (HCV) is a parenterally transmitted virus [5]. Sexual and vertical transmission of HCV is considered inefficient but co-infection of HIV and HCV increases the risk of perinatal transmission of either virus [5].

Between 2 and 20% of HIV-positive individuals in sub-Saharan Africa are also infected with HBV [6]–[8]. The consequences of co-infection are increased liver-related morbidity and mortality, increased viral replication of either virus and, in the context of antiretroviral therapy for HIV (ART), immune reconstitution inflammatory syndrome (IRIS) and hepatotoxicity [9], [10]. In recent years, lamivudine, emtricitabine, and tenofovir have been approved for the management of HIV/HBV co-infection. This raises a number of possibilities and concerns related to the management of these infections. Studies have demonstrated that anti-HBV-active ART makes it possible to achieve suppression of HBV replication in a significant number of co-infected patients [11]. On the other hand, the HBV status of most HIV patients in Africa is not known, which means that many are unknowingly receiving mono-therapy for HBV infection in the context of their ART for HIV. Mono-therapy with lamivudine has been shown to induce HBV resistance in 24% of HBV mono-infected patients after one year, increasing to 71% after 5 years of treatment [12]. Moreover, resistance to lamivudine confers partial or complete cross-resistance to the HBV inhibitors emtricitabine, telbivudine and entecavir, thus limiting future treatment options for HBV infection. In contrast, an ART backbone of tenofovir/emtricitabine or tenofovir/lamivudine could be expected to suppress HBV replication effectively while inducing less HBV resistance [13].

HIV and HCV co-infections are likely to be less common in sub-Saharan Africa due to the differences in transmission routes but only limited data are available [14]. HIV infection, alcohol consumption, and older age at the time of HCV infection have been shown to be associated with a higher rate of liver fibrosis progression [4]. Treatment for HCV with pegylated interferon is rarely available in sub-Saharan African public health care settings.

Data on the prevalence and incidence of HBV and HCV infection in Rwandan HIV-infected adults are scarce. One study among HIV-infected pregnant women in 2007 found a seroprevalence of 2.4% for active HBV and 4.9% for anti-HCV antibodies [15]. Since 2011, the Rwandan government recommends that all HIV patients co-infected with HBV receive an ART regimen containing tenofovir and lamivudine or emitricitabine [16] but HIV patients are not routinely tested for HBV. We observed HIV patients receiving care at a public HIV clinic in Kigali, Rwanda, between 2007 and 2010, and documented various behavioral, clinical, and laboratory endpoints (the SEARCH study). Within this context, we determined the prevalence and determinants of active and past HBV infection and anti-HCV antibodies and the incidence of active HBV infection, assessed the effect of HBV and HCV infection on HIV disease progression, and described the ART regimens that patients with active HBV co-infection are currently receiving.

## Methods

The SEARCH study (Side Effects and Reproductive Health in a Cohort on HAART) was a prospective cohort study in Kigali, Rwanda, to evaluate ART adherence and outcomes, and the impact of ART on various aspects of reproductive and sexual health, including HBV and HCV. The SEARCH study was conducted at the HIV outpatient clinic of the Center for Treatment and Research on AIDS, Tuberculosis and Malaria (TRAC-Plus), which is now part of the Institute of HIV/AIDS, Disease Prevention and Control (IHDPC) within the Rwanda Biomedical Center. Ethical approval was obtained from the Rwandan National Ethics Committee. All study participants provided written informed consent prior to enrollment, were free to withdraw from the study at any time, and continued to receive care within publicly-funded HIV treatment programs at the end of their study participation.

### Study Design and Population

The SEARCH study was designed to enroll 100 women and 100 men initiating ART and 200 women who did not yet qualify for ART according to the Rwanda Ministry of Health guidelines because they had a CD4 count higher than 350 cells/µl and were asymptomatic [16]. All eligible patients attending the TRAC-Plus clinic between November 2007 and January 2010 were offered enrollment into the study. Patients were followed up for 6 to 24 months, until the study was closed in August 2010. Only data collected during the first 12 months of follow-up are presented in this paper. The study visit schedule followed the national ART program schedule as much as possible so that study participants could easily be transferred from SEARCH to the national ART program when the study ended. Women not eligible for ART were seen at baseline and 3-monthly intervals thereafter, and they were switched to the ART cohort when they became eligible for ART. For patients initiating ART, clinic visits were scheduled at baseline (ART initiation) and at week 2 and months 1, 2, 3, 6, 9, and 12 after ART initiation. In addition, pharmacy visits were scheduled monthly.

The study population consisted of ART-naïve HIV patients, 18 years or older, seeking care at the TRAC-Plus clinic. ART eligibility was determined by a TRAC-Plus committee according to the Rwanda Ministry of Health guidelines and clinic protocols. Study eligibility criteria also included residing within travel distance from the TRAC-Plus clinic, and being willing and able to adhere to the study protocol and give informed consent. The main exclusion criteria were pregnancy and diagnosis or clinical suspicion of tuberculosis.

### Study Procedures

All participants were interviewed regularly throughout the study to collect information on demographics, sexual and contraceptive behavior, nutritional status, medical and reproductive history, use of medications, and symptoms (with a focus on those potentially related to HIV infection, ART use or reproductive health problems). Clinical assessments were also carried out at most visits and included a general physical exam, a speculum exam in women, and targeted assessments for neuropathy and lipodystrophy. Blood samples were collected at all clinic visits. They were hand-carried from the clinic to the laboratory next door within 4 hours after collection. CD4+ T-cell counts on EDTA blood samples were always done on the day of sample collection. All other samples were centrifuged to separate plasma from blood cells, aliquoted and stored at −80°C on the day of sample collection until further processing and testing.

All laboratory tests relevant to this paper were carried out at the National Reference Laboratory in Kigali, Rwanda. At study enrollment, the presence of HIV infection in each study participant was confirmed using a 4th generation HIV ELISA. CD4+ T cell counts (FACSCalibur, Becton Dickinson, San Jose, CA, USA) were done every 3 months for women who did not yet qualify for ART and every 6 months for those initiating ART. Plasma HIV RNA viral loads (COBAS AmpliPrep/COBAS TaqMan HIV-1 Test versions 2.0, Roche Molecular Diagnostics, Pleasanton, CA, USA) were done at ART initiation and every 12 months thereafter. The lower limit of detection was 40 HIV RNA copies/ml.

An hCG urine pregnancy test was done every 6 months and testing for various sexually transmitted infections (STIs) every 6 or 12 months. Herpes simplex type 2 (HSV-2) serology was done every 12 months using HerpeSelect test kits (Focus Diagnostics, Cypress, CA, USA). The liver enzymes alanine aminotransferase (ALT) and aspartate aminotransferase (AST) (COBAS Integra Chemistry Analyzer, Roche Diagnostics, Mannheim, Germany) were determined at all visits post ART initiation.

HBV and HCV testing was conducted on all available baseline and Month 12 specimens after data collection had been completed and the study had been closed. About 4 mL plasma in EDTA Vacutainer tubes had been stored at −80°C. After thawing, specimens were tested for HBV surface antigen (HBsAg) using the Murex HBsAg version 3 kit, anti-HBV core antibody (anti-HBc) using the Murex anti-HBc total kit, and anti-HCV IgG antibody (anti-HCV) using the Murex anti-HCV version 4 kit (for all: Abbott Murex, Dartford, UK). However due to a kit shortage, 38 specimens were tested on the Abbott ARCHITECT i2000/SR (Abbott Diagnostics, Lake Forest, IL, USA) using test kits with similar specificity and sensitivity as the Murex kits. All assays were performed according to manufacturer instructions. HBV DNA PCR and HCV RNA PCR were not available in Rwanda at the time of the study. Participants with clinically relevant abnormal test results for HBV and HCV were referred to Kigali University Teaching Hospital, Department of Gastro-enterology for clinical follow-up.

### Statistical Analysis

Data were analyzed using STATA version 11.0 (StataCorp, College Station, TX, USA). Stem and leaf plots and Shapiro-Wilk tests were used to investigate the normality of data distribution. The Pearson Chi-square test, student’s t-test or ANOVA test were used to test for differences between groups in the case of normally distributed data, and non-parametric tests were used otherwise.

A positive test for HBsAg, regardless of anti-HBc test result, was interpreted as active HBV infection (acute or chronic). A positive test for anti-HBc in combination with a negative test for HBsAg is referred to as a past infection in this manuscript, but we could not differentiate past infections from occult infections. Any exposure to HBV was defined as a positive test for anti-HBc with or without a positive HBsAg test. A positive test for anti-HCV antibodies was interpreted as evidence of past or ongoing HCV infection. Incident HBV infection was defined as having a positive HBsAg test at Month 12 among participants with a negative HBsAg test at baseline. Virological failure to ART was defined as having more than 40 HIV RNA copies/mL 12 months after ART initiation.

Potential determinants of HBV infection were assessed in bi- and multivariable models using multinomial logistic regression with active HBV infection (HBsAg positive regardless of anti-HBc result), past HBV infection (anti-HBc positive and HBsAg negative), and never having been HBV exposed (negative for both) as the outcome variable. Potential determinants of anti-HCV antibodies were assessed in bi- and multivariable binary logistic regression models. In all multivariable models, potential determinants were selected based on evidence from published studies (age, gender), Akaike’s information criteria, and a p-value of less than 0.2 in bivariable models. No evidence for collinearity was found in least squares regression models containing the same variables as our logistic regression models (all variance inflation factors were below 2). Sensitivity analyses were conducted to determine the robustness of the models by excluding participants who were anti-HCV positive at baseline from the analyses with HBV as the outcome. This did not alter any of our conclusions and results are therefore not presented.

## Results

**Figure 1:**
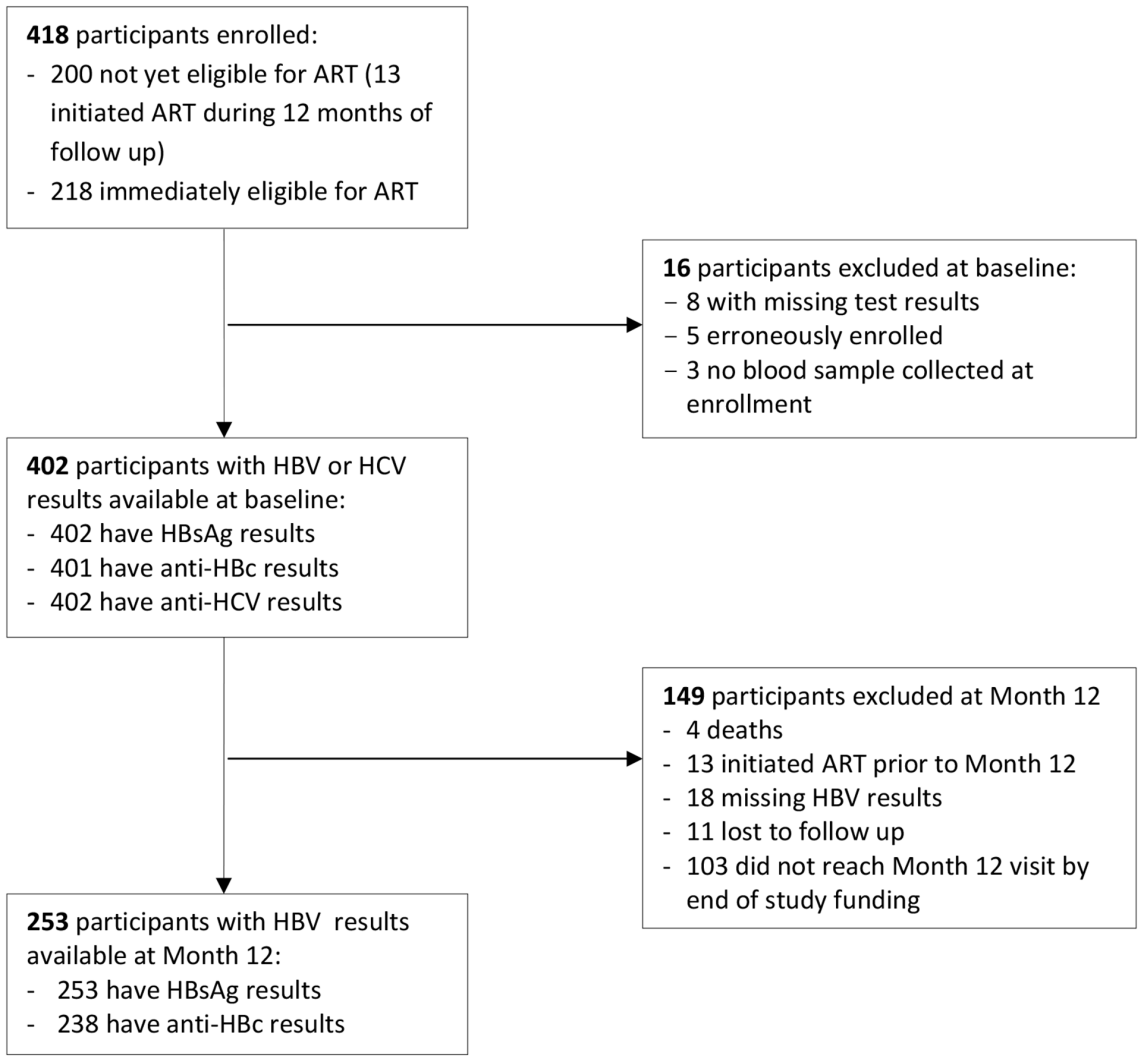
Participant flowchart.

Four hundred and eighteen participants were enrolled into this study: 104 men and 114 women initiating ART, and 200 women who were not yet eligible for ART. A total of 16 participants were excluded from the baseline analyses due to erroneous enrollment or missing blood specimens or test results (Figure 1). During follow-up, 4 participants died, 13 transferred from the pre-ART to the ART group, 11 were lost to follow-up, 103 had not yet reached the Month 12 visit when the study was terminated, and 18 had missing HBV test results at Month 12. The analysis sample therefore consisted of 402 participants at baseline, and 253 of them (63%) had HBV test results at 12 months of follow up.

### Baseline Characteristics

The mean age of study participants was 34.6 years, and those who had never been exposed to HBV were significantly younger than those who had been exposed to HBV or HCV (Table 1). More than half (56.9%) of the participants were married, and most had attended primary (50.2%) or secondary (35.8%) school. Eight percent of the participants reported to have two or more sexual partners, 47.0% used a condom during their last vaginal sex act, and 38.1% regularly consumed alcohol (defined as alcohol use on more than 3 days per week) during the previous 6 months. Injection drug use was not reported by anyone.

**Table 1:** Baseline characteristics of HIV-infected participants by HBV and HCV status.

Baseline Characteristic	Active HBV n(% of 21)	Past HBV n(% of 172)	Never Exp to HBV n(% of 208)	HCV n(% of 23)	Total n(% of 402)
**Mean age yrs (SD)** 1	37.6 (6.7)	35.5 (8.7)	33.6 (7.5)	40.9 (9.7)*	34.6 (8.0)
**Gender**					
Male	7 (33.3)	47 (27.3)	44 (21.2)	4 (17.4)	99 (24.6)
Female	14 (66.7)	125 (72.7)	164 (78.8)	19 (82.6)	303 (75.4)
**Marital status** 1					
Never married	2 (9.5)	17 (10.1)	19 (9.3)	1 (4.5)	38 (9.6)
Married	8 (38.1)	96 (56.8)	120 (58.8)	10 (45.4)	224 (56.9)
Divorced	5 (23.8)	26 (15.4)	26 (12.8)	6 (27.3)	57 (14.5)
Widowed	6 (28.6)	30 (17.7)	39 (19.1)	5 (22.7)	75 (19.0)
**Educational level** 1					
None	2 (10.5)	15 (8.9)	22 (10.7)	4 (18.2)	39 (9.9)
Primary school	6 (31.6)	87 (51.5)	104 (50.7)	11 (50.0)	198 (50.2)
Secondary school	10 (52.6)	58 (34.3)	73 (35.6)	6 (27.3)	141 (35.8)
Post secondary school	1 (5.3)	9 (5.3)	6 (3.0)	1 (4.5)	16 (4.1)
**Median weekly income in USD (IQR)**	12.5 (2.1–20.8)	8.3 (0–20.8)	6.3 (0–16.7)	6.3 (0–12.5)	8.3 (0–20.8)
**Median household size (IQR)**	6 (4–7)	5 (3–7)	4 (3–6)	6 (4–8)	4 (3–6)
**Median age at sexual debut in years (range)** 1	18 (12–31)	18 (13–30)	18 (6–30)	18 (14–30)	18 (6–31)
**≥2 sexual partners last 6 mo** 1	0 (0.0)	18 (11.2)	14 (7.0)	1 (4.4)	32 (8.4)
**Condom use last vaginal sex**	11 (55.0)	83 (49.7)	91 (44.2)	6 (26.1)	185 (47.0)
**Regular alcohol use** 2	11 (52.4)	68 (40.5)	71 (34.5)	5 (21.7)	151 (38.1)
**Body mass index (BMI)** 1					
Normal (18.5–24.9 kg/m^2^)	9 (47.4)*	83 (51.6)*	123 (62.8)*	11 (50.0)	216 (57.3)
Underweight (<18.5)	3 (15.8)	21 (13.0)	13 (6.6)	1 (4.5)	37 (9.8)
Overweight (25–29.9)	2 (10.5)	40 (24.8)	41 (20.9)	8 (36.4)	83 (22.0)
Obese (> = 30)	5 (26.3)	17 (10.6)	19 (9.7)	2 (9.1)	41 (10.9)
**Positive HSV-2 serology**	16 (76.2)	150 (87.7)	176 (85.0)	22 (95.7)	343 (85.8)
**WHO clinical stage** 1					
Stage 1	10 (50.0)*	118 (69.8)*	157 (77.3)*	12 (52.2)	285 (72.5)
Stage 2	5 (25.0)	38 (22.5)	28 (13.8)	7 (30.4)	71 (18.1)
Stage 3	4 (20.0)	12 (7.1)	17 (8.4)	4 (17.4)	33 (8.4)
Stage 4	1 (5.0)	1 (0.6)	1 (0.5)	0 (0.0)	4 (1.0)
**ART groups** 3					
No ART	5 (23.8)	78 (45.3)	113 (54.3)	10 (43.5)	196 (48.8)
AZT/D4T, 3TC, NVP/EFV	14 (66.7)	72 (41.9)	79 (38.0)	10 (43.5)	166 (41.3)
TDF, 3TC, NVP/EFV	2 (9.5)	22 (12.8)	16 (7.7)	3 (13.0)	40 (9.9)
**Median CD4 count cells/µl (IQR)**	276 (195–487)	332 (230–509)	389 (224–535)	359 (220–493)	359 (221–518)
**Median log_10_ HIV RNA copies/mL (IQR)**	4.41 (3.97–5.23)	4.24 (3.25–4.91)	4.38 (3.58–5.00)	4.01* (2.49–4.61)	4.31 (3.43–4.99)

* p<0.05 comparing the 3 HBV groups or comparing anti-HCV positive versus negative.

1 Missing values. Age: n = 397; marital status: n = 394; educational level: n = 394; age at sexual debut: n = 393; sexual partners in last 6 months: n = 379; BMI: n = 377; WHO clinical stage: n = 393.

2 Regular alcohol use was defined as alcohol use more than 3 times per week during the last 6 months.

3 AZT = zidovudine; D4T = stavudine; 3TC = lamivudine; NVP = nevaripine; EFV = efavirenz; TDF = tenofovir diphosphate.

Most participants had a normal body mass index (BMI; 57.3%) and those with an abnormal BMI were more often overweight or obese (32.9%) than underweight (9.8%). The majority of participants (85.8%) had positive HSV-2 serology. Most participants were in WHO clinical stage 1, but the distribution of clinical stage varied significantly between the HBV and HCV outcome groups (see Table 1 and further). All participants who initiated ART at baseline and who had a Month 12 follow-up visit were given a first-line regimen that contained lamivudine but not tenofovir, which was in agreement with the national treatment guidelines at that time (Table 1). Participants initiating ART had a baseline median CD4 count of 222 cells/µl (interquartile range (IQR) 142–289) and a median HIV RNA viral load of 4.81 log_10_ copies/ml (IQR 4.22–5.33). Participants not initiating ART had a baseline median CD4 count of 513 cells/µl (IQR 413–651) and a median HIV RNA viral load of 3.69 log_10_ copies/ml (IQR 2.92–4.41).

### Prevalence and Incidence of HBV and HCV

The baseline prevalence of active HBV was 5.2% (21/402; 95% CI 3.0–7.4), past HBV 42.9% (172/401; 95% CI 38.0–47.8) and never having been exposed to HBV 51.9% (208/401; 95% CI 47.0–56.7). Of the 21 cases of active HBV at baseline, 6 were HBsAg-negative at Month 12, 10 were still HBsAg-positive, and 5 were not retested. Between baseline and Month 12, one new case of active HBV was detected, giving an incidence rate of 4.2/1,000 person years of follow-up (PY) (95% CI 0/1,000–12.5/1,000 PY). The baseline prevalence of anti-HCV was 5.7% (23/402; 95% CI 3.44–8.0%). At baseline, 3 participants tested positive for both HBsAg and anti-HCV and 12 for both anti-HBc and anti-HCV.

### Baseline Determinants of HBV and HCV Infection

In the multivariable model, participants with active HBV infection presented with a more advanced WHO clinical stage (stage 3 or 4 compared to stage 1; adjusted RR 4.19; 95% CI 1.21–14.47) than participants who had never been exposed to HBV (Table 2). Participants with past HBV infection were more likely to be older than those who had never been exposed to HBV with a 3% increased risk for each year increase in age (adjusted RR 1.03; 95% CI 1.01–1.06). Baseline CD4 counts and HIV viral loads were not significantly different between the three HBV outcome groups (Tables 1 and 2).

**Table 2:** Determinants of HBV and HCV infection at baseline.

	Crude^1^			Adjusted^2^		
	RR	95% CI	p	RR	95% CI	p
**Active HBV (n = 21) compared to never exposed (n = 208)**						
Age in years	1.06	(1.01–1.22)	0.03	1.05	(0.98–1.12)	0.10
Female vs male	0.54	(0.20–1.41)	0.20	0.65	(0.19–2.22)	0.49
WHO stage 2 vs 1	2.80	(0.89–8.82)	0.08	2.27	(0.63–8.19)	0.20
WHO stage 3,4 vs 1	4.36	(1.34–14.18)	0.01	4.19	(1.21–14.47)	0.02
Baseline CD4 cells/µl 200–350 vs >350	1.63	(0.54–4.95)	0.39	0.83	(0.23–3.13)	0.79
<200 vs >350	2.13	(0.70–6.54)	0.18	0.88	(0.22–3.47)	0.85
**Past HBV (n = 172) compared to never exposed (n = 208)**						
Age in years	1.03	(1.00–1.06)	0.03	1.03	(1.01–1.06)	0.02
Female vs male	0.71	(0.44–1.44)	0.16	0.79	(0.44–1.40)	0.42
WHO stage 2 vs 1	1.81	(1.05–3.11)	0.03	1.70	(0.95–3.06)	0.08
WHO stage 3,4 vs 1	0.96	(0.45–2.04)	0.92	0.81	(0.37–1.79)	0.60
Baseline CD4 cells/µl 200–350 vs >350	1.36	(0.84–2.21)	0.21	1.14	(0.66–1.96)	0.63
<200 vs >350	1.25	(0.73–2.14)	0.42	0.95	(0.50–1.82)	0.90
**Anti-HCV (n = 23) compared to no anti-HCV (n = 379)**						
Age in years	1.10	1.04–1.16	<0.001	1.10	1.05–1.17	<0.001
Female vs male	1.56	0.53–4.78	0.41	1.45	0.43–4.92	0.55
WHO stage 2 vs 1	2.49	0.94–6.57	0.06	2.49	0.87–7.19	0.09
WHO stage 3,4 vs 1	2.76	0.84–9.04	0.09	4.05	1.12–14.58	0.03
Baseline HIV RNA (log_10_ copies/ml)	0.68	0.48–0.95	0.03	0.60	0.40–0.98	0.01

1 The following variables were also considered for their association with HBV and HCV but were not statistically significant: multiple sexual partners, age at sexual debut, condom use, weekly income, regular alcohol use, positive HSV2 serology, and baseline HIV viral load. BMI was not included in any of these models because of the high likelihood of reverse causality.

2 The multivariable models contained all variables listed in the table for each outcome.

In the multivariable HCV model, a year increase in age led to a 10% increased odds of being anti-HCV positive (adjusted OR 1.10, 95% CI 1.05–1.17) (Table 2). A more advanced WHO clinical stage was positively associated with anti-HCV (stage 3 or 4 compared to stage 1; adjusted OR 4.05; 95% CI 1.12–14.58) but a higher baseline HIV viral load was negatively associated with anti-HCV (adjusted OR 0.60; 95% CI 0.40–0.98).

### ART Regimens Taken by HIV-HBV Co-infected Patients

Among those with active HBV at baseline (n = 21), 16 were in the group that initiated ART immediately: 14 initiated an ART regimen containing lamivudine but not tenofovir and 2 initiated a regimen including tenofovir. Only the 14 patients not using tenofovir were retested at Month 12∶4 were HBsAg-negative and anti-HBc positive at Month 12 and 10 developed persistent infection. One person switched to tenofovir at the Month 12 visit but this switch was related to drug toxicity and not to the patient’s HBV status. Of the 5 HIV-HBV co-infected patients who did not initiate ART at baseline, only 2 were retested at Month 12. Both of them were HBsAg-negative and anti-HBc positive at Month 12.

### Effects of HBV and HCV on Liver Enzymes and HIV Disease Progression

Liver enzymes were monitored only after participants had initiated ART. No statistical differences in median ALT and AST levels at baseline and Month 12 were found between the different HBV and HCV outcome groups (Table 3).

**Table 3:** Liver enzymes in HBV and HCV infected patients initiating ART^1^.

	Active HBV		Past HBV		Never Exposed to HBV		HCV
	Baseline n = 16	M12 n = 4	Baseline n = 81	M12 n = 52	Baseline N = 81	M12 n = 62	Baseline n = 11
**Median ALT (IQR**)	23.5 (16.8–40.9)	15.6 (10.8–24.7)	19.3 (13.0–24.7)	23.2 (16.3–32.6)	22.0 (15.5–32.9)	21.2 (15.4–31.0)	31.6 (20.6–45.6)
Grade 1^2^	1 (6.3)	0	0	1 (1.9)	2 (2.5)	1 (1.61)	0
Grade 2	0	0	0	0	1 (1.2)	0	0
Grade 3	0	0	1 (1.2)	0	0	0	0
Grade 4	0	0	0	0	0	0	0
**Median AST (IQR)**	32.1 (27.4–52.2)	21.4 (18.7–25)	26 (21.2–34.7)	26.2 (21.1–32)	27.9 (22.8–36.4)	27.1 (21–32.1)	28.8 (23.3–59.4)
Grade 1^2^	1 (6.3)	0	1 (1.3)	1 (2.3)	1 (1.3)	1 (1.83)	0
Grade 2	0	0	0	0	1 (1.3)	0	0
Grade 3	0	0	1 (1.3)	0	0	0	0
Grade 4	0	0	0	0	0	0	0

1 ALT and AST only collected for patients initiating ART (n = 210).

2 According to the DAIDS Table for Grading the Severity of Adult and Pediatric Adverse Events (version December 2004): Grade 1 (1.25–2.5 ULN), Grade 2 (2.51–5 ULN), Grade 3 (5.1–10 ULN), Grade 4 (>10 ULN).

Among those who had initiated ART, participants with active HBV at baseline achieved a median CD4 increase of only 28 cells/µl/year while participants who had never been exposed to HBV achieved a median increase of 113 cells/µl/year (p = 0.04). There was no statistically significant difference in the median CD4 increase between participants who had been infected with HBV in the past (127 cells/µl/year) and those who had never been exposed (113 cells/µl/year). Among those who had initiated ART, 69% of participants with active HBV or past HBV exposure at baseline achieved full HIV viral load suppression (defined as HIV RNA levels below 40 copies/ml) after 12 months of ART compared to 83% of the participants who had never been exposed to HBV (p = 0.11). In a multivariable analysis controlled for baseline CD4 count and baseline HIV viral load, participants with active HBV infection or past exposure to HBV at baseline had 2.40 increased odds of virological failure compared to non-HBV exposed participants (OR 2.40; 95% CI 1.04–5.51).

Participants with anti-HCV at baseline had a slower CD4 count recovery rate of 58 cells/µl/year compared to participants without anti-HCV (126 CD4 cells/µl/year; p = 0.07). HIV virological failure did not differ significantly between participants with anti-HCV (60%) and those without (76%; p = 0.26).

## Discussion

The prevalence rates of active HBV infection and anti-HCV in this cohort of HIV patients seeking care in an urban HIV clinic in Rwanda were high at 5.2% and 5.7%, respectively. Almost half of all patients (42.9%) had ever been exposed to HBV. These rates are slightly higher than those reported in 2007 for HIV-positive pregnant women in Rwanda (2.4% for active HBV and 4.9% for anti-HCV) [15]. High rates have also been found in other African settings [17]–[24]. In fact, several African studies have found even higher rates for HBsAg prevalence in HIV-positive patients than we have [22]–[24].

The major limitation of our study is that we could not identify occult HBV infections in those who were HBsAg-negative but anti-HBc-positive by HBV DNA PCR testing, and that we could not identify active HCV infections among those positive for anti-HCV by HCV RNA PCR testing. Occult active HBV infections are thought to be common among HIV patients, with reported prevalence rates ranging from 10–14% [25]–[27] to 89% in one study in South Africa [20]. Given the fact that 42.9% of HIV patients in Rwanda had isolated anti-HBc, further research is needed to determine the prevalence of occult HBV in this group. Similarly, further research is needed to determine the prevalence of active HCV infection among those with anti-HCV. About 25% of HCV infections clear spontaneously (resulting in a positive anti-HCV test but a negative HCV RNA PCR test) [28]. Furthermore, while anti-HCV testing has been used extensively to estimate HCV prevalence and incidence, recent studies comparing antibody tests with HCV RNA PCR suggest that false-positive antibody test results are common in African settings [29]. Unfortunately, routine confirmation of positive anti-HCV test results with HCV RNA PCR is expensive and not feasible in many African settings. Larger studies designed to determine the sensitivity and specificity of HCV assays, and to evaluate affordable diagnostic algorithms, in African settings are urgently needed.

Other limitations of our study included the fact that ALT and AST were only evaluated in patients after they had initiated ART and the fact that 37% of the patients had less than 12 months follow-up due to study closure. A strength of our study is that we worked within a public HIV care setting and are therefore reporting results that are relevant to local policymakers.

In our study, older age was associated with past exposure to HBV infection and with exposure to HCV infection. This most likely reflects ongoing exposure over time, as has been reported by others [17], [21]. Gender was, however, not associated with active HBV, past HBV or anti-HCV in our study, which is in contrast with several other studies that have shown strong associations with male gender [17], [19], [20], [23]. This could be explained by the fact that our study oversampled women by design and did not include any injecting drug users. We did not find any associations between HBV and sexual risk behaviors, which is also in contrast with other studies [1], [2], [18]. In our cohort, sexual risk taking was modest, with 8.2% of the participants reporting multiple partners and 47.0% reporting to have used a condom during the last sex act. We did not ask the male participants whether they have had sex with other men.

WHO clinical stage 3 or 4 at baseline was associated with a higher likelihood of having an active HBV or anti-HCV at baseline, but while the median baseline CD4 count was lower among those with active HBV (but not among those with anti-HCV), this was not statistically significant. However, having active HBV or anti-HCV at baseline were both associated with a slower CD4 count recovery rate during follow-up. This has also been found in a study in Ghana [30], but not in studies in South Africa, Nigeria, and Denmark [31]–[33]. Surprisingly, having a detectable HIV RNA viral load at baseline was less likely in those who had HCV antibodies at baseline. This has not been confirmed by other studies [34], and should be interpreted with caution because we only conducted anti-HCV testing and did not confirm infections with HCV RNA PCR. HIV virological failure after 12 months of ART was more likely in those with active HBV at baseline but not in those with anti-HCV. Reports in the literature about virological failure rates in those with and without active HBV are conflicting [31]–[33]. Finally, we did not find any statistical differences in median ALT and AST levels between the different HBV and HCV outcome groups, but the comparison groups were small. Reports in the literature in this area are also conflicting [20]–[22], [30], [31]. Additional carefully designed studies investigating the impact of HBV and HCV co-infection on ART response and on hepatotoxicity are therefore needed.

During the first 12 months of the study, almost all participants on ART were using a regimen containing lamivudine but not tenofovir, which was in accordance with the Rwanda antiretroviral treatment guidelines for HIV infection prior to the revision in 2011. Of the participants with HIV and active HBV co-infection who were on ART, 88% were receiving lamivudine monotherapy for their HBV infection. Studies have shown that treatment with two anti-HBV-active drugs simultaneously reduces the emergence of drug resistance [11]–[13].

In conclusion, active HBV infection and anti-HCV are both common in HIV-infected patients in Rwanda and are associated with a slower rate of CD4 recovery in the context of HIV-suppressive ART. Our study confirms the importance of screening HIV patients in Rwanda for HBsAg to ensure that HIV-HBV co-infected patients receive an HBV-active ART regimen. The prevalence of occult HBV infection should be determined and improved access to HBV vaccination considered. Active HCV prevalence and incidence should be investigated further to determine whether HCV RNA PCR testing should be introduced in Rwanda.
